# Glycerol conversion to 1, 3-Propanediol is enhanced by the expression of a heterologous alcohol dehydrogenase gene in *Lactobacillus reuteri*

**DOI:** 10.1186/2191-0855-1-37

**Published:** 2011-11-04

**Authors:** Hema Vaidyanathan, Vijayalakshmi Kandasamy, Gopi Gopal Ramakrishnan, KB Ramachandran, Guhan Jayaraman, Subramanian Ramalingam

**Affiliations:** 1 Centre for Biotechnology, Anna University, Chennai 600 025, Tamil Nadu, India; 2 Department of Biotechnology, Indian Institute of Technology Madras, Chennai 600036, Tamil Nadu, India

**Keywords:** 1, 3-propanediol oxidoreductase, YqhD, NADPH, 3-HPA, *L. reuteri*

## Abstract

In this work, *Lactobacillus reuteri *has been metabolically engineered for improving 1, 3-propanediol (1, 3-PD) production by the expression of an *Escherichia coli *alcohol dehydrogenase, *yqhD*, that is known to efficiently convert the precursor 3-hydroxypropionaldehyde (3-HPA) to 1, 3-PD. The engineered strain exhibited significantly altered formation rates for the product and other metabolites during the fermentation. An increase in the 1, 3-PD specific productivity of 34% and molar yield by 13% was achieved in the clone, relative to the native strain. A concomitant decrease in the levels of toxic intermediate, 3-HPA, was observed, with the specific productivity levels being 25% lesser than that of the native strain. Interestingly, the recombinant strain exhibited elevated rates of lactate and ethanol formation as well as reduced rate of acetate production, compared to the native strain. The preferential utilization of NADPH by YqhD with a possible decrease in the native 1, 3-PD oxidoreductase (NADH-dependent) activity, could have resulted in the diversion of surplus NADH towards increased lactate and ethanol productivities.

## Introduction

Biological processes are eco-friendly and sustainable alternatives to conventional chemical processes for production of several industrially important bulk chemicals like succinic acid, lactic acid, 1, 3-propanediol, 1, 4-butanediol, etc. (Biebl et al. 1998; Chotani et al. 2000; Song and Lee 2006). Such processes could be economically viable if they are based on renewable feedstocks. Glycerol, a surplus byproduct of the biodiesel industry holds promise as a major feedstock for synthesis of platform chemicals such as 1, 3-propanediol (Zhu et al. 2002). Currently, 1, 3-propanediol (1, 3-PD) has attracted worldwide interest due to its enormous applications in polymers, cosmetics, foods, adhesives, lubricants, laminates, solvents, antifreeze and medicines (Homann et al. 1990; Colin et al. 2000; Zhu et al. 2002; Cheng et al. 2007).

The biological route involves 1, 3-PD production by microorganisms like *Klebsiella, Citrobacter, Enterobacter, Clostridia *and *Lactobacilli *(Biebl et al. 1999; Saxena et al. 2009). Amongst these, *Clostridium butyricum *and *Klebsiella pneumoniae*, are considered to be the best producers (Gonzalez-Pajuelo et al. 2006). 1, 3-PD concentrations in the range of around 40 - 100 g/l have been obtained with these producers (Celinska 2010). The product levels of the native producers have been improved using various bioprocess strategies. Metabolic engineering is currently being attempted to further enhance the product levels (Saxena et al. 2009).

The non-native producers, *Escherichia coli *and *Saccharomyces cerevisiae*, have also been engineered for 1, 3-PD production. In *S. cerevisiae*, due to ineffective transport of vitamin B12 needed for 1, 3-PD synthesis, only low levels of the product has been obtained. On the other hand, *E. coli *has been metabolically engineered by DuPont and Genencor International, Inc., to produce 1, 3-PD at a concentration of 135 g/l, (Maervoet et al. 2011) the highest reported so far in the industry. A major concern with the existing 1, 3-PD producers is that a majority of them are opportunistic pathogens, that are less suitable for niche applications in food, cosmetic and biomedical industries. In this context *Lactobacillus reuteri*, a GRAS (generally regarded as safe) organism, offers immense potential as a host for 1, 3-PD production.

**Figure 1:**
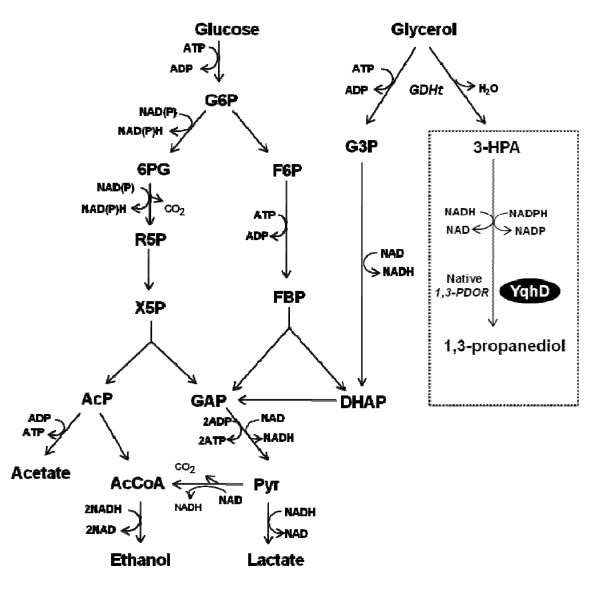
**Pathways of glucose and glycerol metabolism in *L. reuteri***. *Abbreviations*: G6P glucose-6-phosphate, 6PG 6-phosphogluconate, R5P ribulose-5-phosphate, X5P xylulose-5-phosphate, AcP acetyl phosphate, AcCoA acetyl-CoA, F6P fructose-6-phosphate, FBP, fructose-1, 6-bisphosphate, DHAP dihydroxyacetone phosphate, GAP glyceraldehyde-3-phosphate, Pyr pyruvate, G3P glycerol-3-phosphate, 3-HPA 3-hydroxypropionaldehyde, GDHt glycerol dehydratase, 1, 3-PDOR 1, 3-propanediol oxidoreductase in *L. reuteri*, YqhD *E. coli *alcohol dehydrogenase.

*Lactobacillus reuteri *converts glycerol to 1, 3-PD in a two-step anaerobic process (Figure 1). In the first step, a cobalamin-dependent glycerol dehydratase catalyzes the conversion of glycerol to 3-hydroxypropionaldehyde (3-HPA). In the second step, 3-HPA is reduced to 1, 3-PD by a NADH-dependent oxidoreductase (Talarico et al. 1990). 1, 3-PD productivity of around 10-30 g/l has been achieved so far in native *L. reuteri *(Baeza-Jimenez et al. 2011; Tobajas et al. 2009).

The major bottleneck limiting 1, 3-PD production in *L. reuteri *is growth inhibition by secreted metabolites and toxic 3-HPA. These metabolites are produced to regenerate the cofactors such as NADH/NADPH. Therefore redirecting flux from these competing pathways towards product formation by balancing the redox potential would be a powerful metabolic engineering strategy. For instance, disruption of ethanol synthesis has been demonstrated to substantially improve flux through the 1, 3-PD biosynthetic pathway in *K. pneumoniae *(Zhang et al. 2006). Further, redirection of flux from central carbon metabolism towards 1, 3-PD synthesis should be complemented by adequate levels of enzymes and cofactors involved in the pathway.

In this work, we have expressed an *E. coli *alcohol dehydrogenase, *yqhD*, in *L. reuteri*, to increase 1, 3-PD productivity by improved conversion of 3-HPA. Further, the impact of the heterologously expressed *yqhD *on cell growth, 1, 3-PD production and byproduct formation has been analyzed.

## Materials and methods

### Strains and plasmids

The bacterial strains and plasmids used and modified in this study are listed in Table 1.

**Table 1:** Bacterial strains and plasmid vectors used in this work

Strain or plasmid	Description	Source or reference
*E. coli *DH5α	Cloning host for TA vector	Invitrogen, USA
*E. coli *EC1000	Cloning host for pSIP411	Dr Jan Kok, University of Groningen, Netherlands
RBC- TA vector	TA cloning vector	RBC Bioscience Corp., Taiwan
pSIP411	*E. coli*-lactobacillus shuttle expression vector	Sørvig et al. (2005)
*L. reuteri *ATCC55730	Host	Biogaia, Sweden
*L. reuteri *HR2	*L. reuteri *with *yqhD*	This study
*E. coli *K-12 MG1655	Source of *yqh*D gene	Prof. Takashi Horiuchi, National Institute for Basic Biology, Japan.
pHR1	TA vector with *yqhD*	This study
pHR2	pSIP411 with *yqh*D	This study

### Media and growth conditions

*L. reuteri *ATCC 55730 and the *E. coli *strains were grown at 37°C in MRS (MRS contains 5 g yeast extract, 10 g proteose peptone, 10 g beef extract, 2 g dipotassium phosphate, 2 g ammonium citrate, 5 g sodium acetate, 100 mg magnesium sulphate, 50 mg manganese sulphate, 1 g polysorbate 80 and 20 g dextrose, per liter) broth and LB broth, respectively. The recombinants were cultured in media containing appropriate antibiotics, ampicillin (100 μg/mL) and erythromycin (200 μg/mL for *E. coli *and 5 μg/mL for *L. reuteri*). Growth was monitored by measuring the absorbance at 600 nm. Cell dry weight (CDW) was calculated from a predetermined relationship between *L. reuteri *CDW and optical density (1 OD_600 _corresponded to 0.33 g/l CDW).

### Chemicals and Reagents

The enzymes and reagents used in cloning experiments - *Nco*I, *Xho*I, T4 DNA ligase, and Phusion™ Flash High-Fidelity PCR Master Mix - were bought from New England Biolabs (Manassas, USA). Plasmid miniprep spin kit and PCR purification kit were procured from Qiagen (Germany). Primers were procured from VBC-Biotech (Austria) and the inducer sakacin P induction peptide (SppIP) was synthesized from GenScript (USA). Culture media (LB and MRS), the antibiotics, erythromycin and ampillicin, and other chemicals were purchased from HiMedia Laboratories (Mumbai, India). Since 3-HPA standard could not be commercially procured, it was synthesized in our laboratory as described under "3-HPA production by resting cells of *L. reuteri *ATCC 55730".

### Construction of the recombinant plasmids

**Figure 2:**
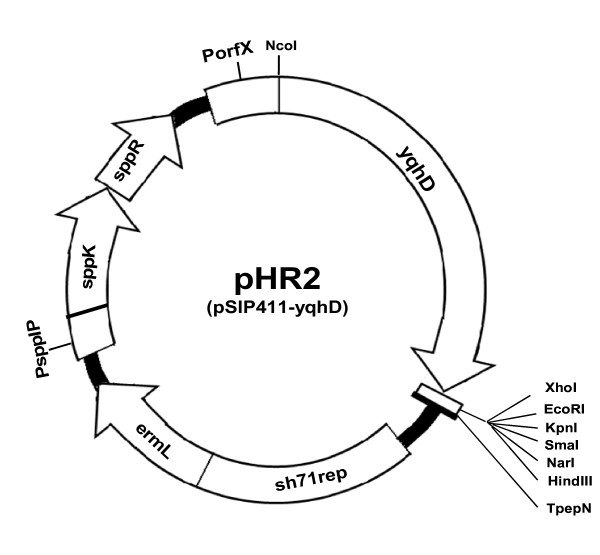
**Structure of the recombinant plasmid pHR2 (~6.86 kb)**. *yqhD E. coli *alcohol dehydrogenase gene, open rectangle MCS, TpepN transcription terminator, sh71rep replication origin for Lactobacillus, ermL erythromycin-resistance marker, PssIP and PorfX inducible promoters, sppK and sppR histidine protein kinase and response regulator respectively.

A schematic representation of the structure of recombinant plasmid, pHR2, carrying *yqhD*, is shown in Figure 2. The 1.163 kb *yqhD *gene fragment (GenBank accession number NC010498), was amplified from the chromosomal DNA of *E. coli *K-12 MG1655 using the primers yqhDF and yqhDR (Table 2). PCR conditions employed were - an initial denaturation at 98°C (10 s), followed by 25 cycles of the program: 98°C (3 s); 65°C (5 s); 72°C (20 s) and a final extension at 72°C (1 min).

**Table 2:** Primers and peptide sequences used in this work

Primer name	**Primer sequence**^**a**^
yqhDF (Forward)	5'-CATG CCATGG ACAACAACTTTAATCTGCACACC-3'
yqhDR (Reverse)	5'-CCG CTCGAG TTAGCGGGCGGCTTC-3'
PorfXF (Forward) SppIP	5'-TGAAAATTGATATTAGCG-3' MAGNSSNFIHKIKQIFTHR

^a ^The restriction sites in the primers *Nco*I (forward) and *Xho*I (reverse) have been underlined

The amplicon was cloned into TA vector to generate the recombinant plasmid pHR1. Further, the *yqhD *gene was sub-cloned from pHR1 into *Nco*I/*Xho*I site of pSIP411, resulting in recombinant plasmid, pHR2. The clones were screened by lysate PCR using the primer pair PorfXF and yqhDR (Table 2). The plasmid pHR2 was electroporated into *L. reuteri *to yield, *L. reuteri *HR2. The electrocompetent cells were prepared as described by Berthier et al. (1996). Electroporation was performed with a BTX electroporator, using pulse settings of 1.5 kV, 800 Ω and 25 μF and a time constant of 11 - 13 ms was obtained. The cells were plated on MRS agar containing the required antibiotic and incubated for 24 - 36 h at 37°C until visible colonies were observed. The recombinant plasmid pHR2 was isolated from *L. reuteri *HR2 using the plasmid miniprep kit, with the following modifications: The cells in resuspension buffer, were lysed with 30 mg/mL lysozyme (USB) and incubated at 37°C for 30 minutes. The rest of the procedure was as per the miniprep manual (Qiagen).

### Batch fermentation

The inoculum for the batch reactor was grown in 150 mL MRS broth with erythromycin at 37°C until an OD_600 _of 0.8 - 1.0 was reached. The seed was then inoculated into a 2 L fermentor (KLF 2000 - Bioengineering AG, Switzerland) filled with 1.2 L MRS medium containing erythromycin and glycerol (278 mM). A glucose to glycerol ratio of 1:2.5 has been used in this study for elevated 1, 3-PD synthesis (Tobajas et al. 2009). Fermentation was carried out at 37°C and 250 rpm, in an anaerobic condition. The pH was maintained at 5.5 by the addition of 1.5 M NaOH or 1.5 M H_3_PO_4 _(El-Ziney et al. 1998). The anaerobic condition was established by flushing with sterile nitrogen. At 0.8 OD_600_, the culture was induced with 50 ng/mL of sakacin P induction peptide (SppIP). Samples were removed periodically for determining OD_600_. The culture pellet and supernatant were stored at - 20°C, to be used later for protein and metabolite analyses respectively.

### Substrate and Metabolite Analyses

Concentrations of glucose, glycerol, 1, 3-PD, ethanol, lactate, 3-HPA and acetate in the culture broth were determined using a HPLC (Shimadzu LC-10AT VP) that was equipped with a refractive index detector (RID) and an aminex HPX-87H column (300 × 78 mm, Bio-Rad, USA). The mobile phase consisted of acetonitrile and water in a ratio of 35:65 in 5 mM H_2_SO_4_, at 0.4 mL/min. The temperature of column and RID was maintained at 30°C and 50°C respectively. Samples were filtered through 0.22 μm filters before analysis. 3-HPA standard was synthesized in the lab using resting cells of *L. reuteri *ATCC 55730 as explained below. Quantitation of 3-HPA was done by HPLC, as described by Spinler et al. (2008).

### 3-HPA production by resting cells of *L. reuteri *ATCC 55730

3-HPA was produced as described previously (Spinler et al. 2008; Luthi-Peng et al. 2002). Briefly, *L. reuteri *was cultured in 100 mL MRS broth, incubated anaerobically at 37°C for 24 h. The anaerobic condition was maintained by sparging with nitrogen. The culture was centrifuged and the pellet washed with 50 mM sodium phosphate buffer (pH 7.4). The cells were resuspended in 250 mM glycerol to a concentration of ~1.5 × 10^10 ^cells/mL and incubated anaerobically at 37°C for 2 h. After the 2 h incubation, the culture was pelleted and the 3-HPA-containing supernatant was collected and filter-sterilized using a 0.22 μm filter and the filtrate used for HPLC analysis.

### SDS-PAGE analysis of *yqhD *expression in *L. reuteri*

The SDS-PAGE was conducted on a 12% polyacrylamide gel (Laemmli 1970). The proteins on the gel were stained with 0.025% (w/v) Coomassie Brilliant Blue G-250. Protein concentration was determined by the Bradford method (Bradford 1976) with bovine serum albumin (BSA) as standard.

## Results

### Heterologous expression of alcohol dehydrogenase (*yqhD*) in *Lactobacillus reuteri *ATCC 55730

**Figure 3:**
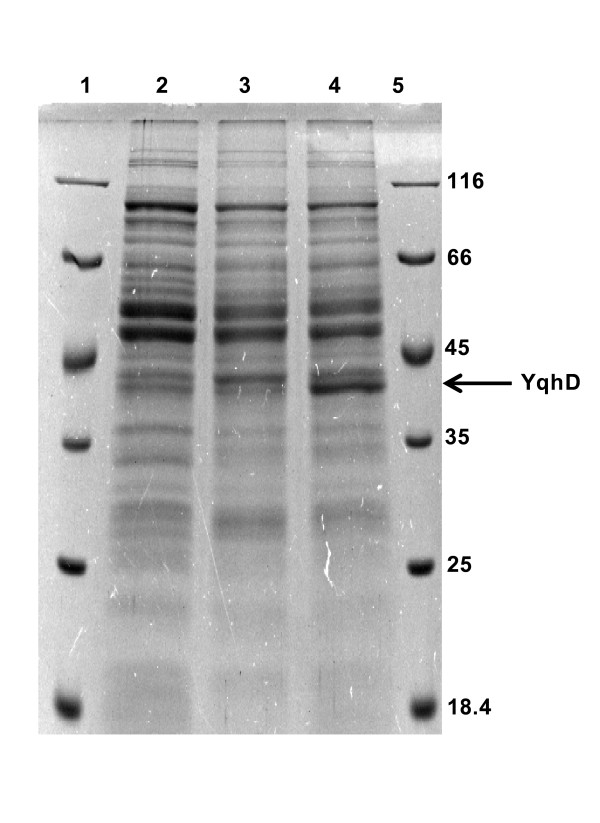
**SDS-PAGE analysis of *L. reuteri *whole cell lysates for *yqhD *expression**. Lane 2 untransformed *L. reuteri*, lane 3 uninduced recombinant *L. reuteri *HR2, lane 4 recombinant 5 h after induction with SppIP, lanes 1 & 5, protein molecular weight marker.

The *E. coli *alcohol dehydrogenase gene (*yqhD*) was cloned and expressed in *L. reuteri*. The recombinant plasmid, pHR2 with *yqhD *gene was constructed as shown in Figure 2. The expression of the cloned *yqhD *gene in *L. reuteri *was confirmed using SDS-PAGE analysis of whole cell lysates (Figure 3). A prominent band of ~43 kDa appeared in the recombinant cells after induction, which correlates well with the expected size of YqhD.

### Batch fermentation analysis of recombinant *L. reuteri *harbouring *yqhD*

**Figure 4:**
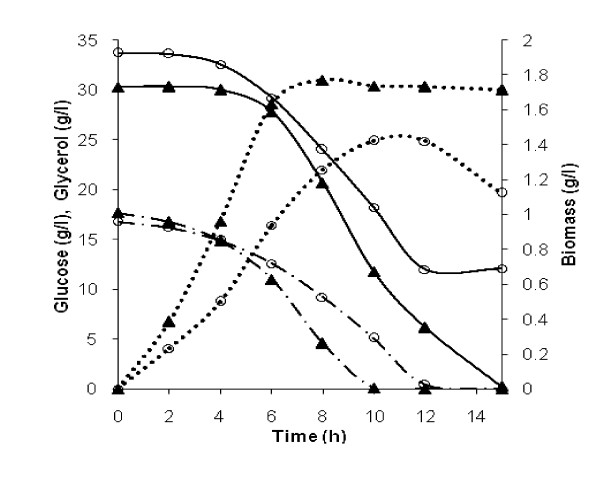
**Time course of glucose (• ― •), glycerol (―) consumption and biomass (••••) growth in native (triangles) and recombinant (open circles) *L. reuteri *strains during batch cultivation**.

To investigate the impact of *yqhD *expression on cell growth, substrate consumption, formation of 1, 3-PD, 3-HPA and other metabolites, batch fermentation of recombinant *L. reuteri *was carried out, with native strain as control. The cell concentration of both native and recombinant strains reached around 1.8 and 1.4 g/l of CDW respectively. The specific growth rate (μ_max_) of the recombinant strain was lower (0.38 h^-1^) compared to the wild type (0.46 h^-1^) (Figure 4).

**Figure 5:**
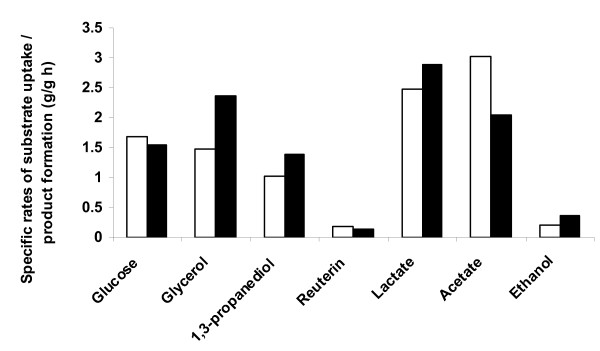
**Specific rates of substrate uptake and product formation in the logarithmic phase of batch fermentation using native (white bar) and recombinant *Lactobacillus reuteri *(black bar) strains**.

It was observed that *yqhD *expression in *L. reuteri*, altered the specific substrate uptake, product and byproduct formation rates significantly (Figure 5). The specific production rate of 1.38 g/g h for 1, 3-PD in the recombinant strain achieved during the log phase after induction, was notably higher (by 34%) than that of the native strain (1.03 g/g h) (Figure 5). This correlates with a 25% decrease in the levels of 3-HPA secreted in the recombinant culture (0.14 g/g h), relative to the native strain (0.19 g/g h) (Figure 5). This enhanced 3-HPA conversion has supposedly contributed to the increased molar yield of 1, 3-PD (up by 13%) observed in the clone (Table 3). Interestingly, the specific rates of formation of lactate and ethanol were higher and that of acetate lower in the recombinant culture, relative to the native strain, during the second half of the logarithmic phase (Figure 5).

**Table 3:** Comparison of 1, 3-PD molar yield of wild type and recombinant *L. reuteri *in batch fermentation

	Glycerol consumed (g/l)	1, 3-propanediol produced (g/l)	Molar yield (mol/mol)
*L. reuteri *ATCC 55730	30.02	11.0	0.45
*L. reuteri *HR2	21.6	9.1	0.51

**Figure 6:**
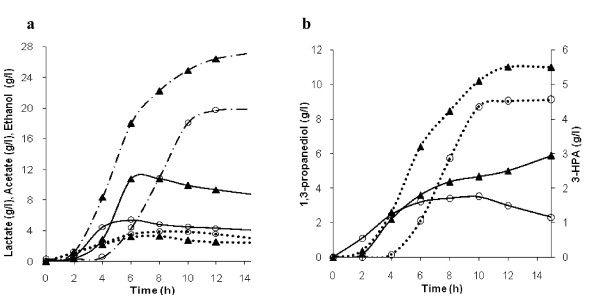
**Time course of metabolite formation by recombinant (open circles) and native strain (triangles) strains of *L. reuteri *in batch cultivation**. **a **lactate (• ― •), acetate (―) and ethanol (••••). **b **1, 3-propanediol (••••) and 3-HPA (―).

The batch experiment has revealed that 1, 3-PD, acetate and ethanol are growth-associated in both the native and recombinant *L. reuteri *strains, while lactate and 3-HPA are growth-associated only in the recombinant strain (Figure 6a, b). During the glucose-glycerol cofermentation, consumption of these two carbon sources was not synchronous. Glucose was consumed more rapidly than glycerol during the early log phase and was exhausted before glycerol in both the native and recombinant strains (Figure 4). In the recombinant strain, glycerol is not utilized upon exhaustion of glucose, while the native strain exhibited moderate glycerol consumption and concomitant 3-HPA synthesis even after depletion of glucose (Figure 4, 6b). However, 1, 3-PD synthesis is observed only when both the carbon sources are utilized in the recombinant and in the native strains during the late-log and early-stationary phase (Figure 4, 6b)

## Discussion

*L. reuteri *produces 1, 3-PD along with 3-HPA only when glycerol is cofermented with glucose. Lower glucose levels have been shown to favour 3-HPA formation. Higher glucose concentrations generate more NADH, that is consumed for reducing 3-HPA to 1, 3-PD. Glycerol serves as an electron sink by recycling NADH produced during glycolysis (Luthi-Peng et al. 2002; Schutz and Radler 1984). In this work, 1, 3-PD synthesis is observed both in native and recombinant strains only when both the carbon sources are utilized (Figure 4, 6b). In the case of native strain, glycerol consumption upon exhaustion of glucose resulted in 3-HPA accumulation, since NADH supply could be limited by reduced glycolysis. Thus redox balance plays a crucial role in 1, 3-PD formation.

Enhancing the enzyme concentration and cofactor availability could lead to improved 1, 3-PD formation. As the phosphoketolase pathway prevalent in *L. reuteri *(Årsköld et al. 2008), provides increased NADPH, overexpression of *yqhD*, has the potential to further improve 1, 3-PD productivity. In this work, expression of *yqhD *has increased the molar yield of 1, 3-PD from glycerol by 13% in *L. reuteri *HR2. This is in contrast to the results reported by Zhuge et al. (2010) in recombinant *K. pneumoniae *strain, wherein *yqhD *overexpression did not increase the 1, 3-PD yield. However, upon overexpression of *yqhD*, they have observed a reduction in the activity of the native 1, 3-PD oxidoreductase (1, 3 PDOR), with increased ethanol production. A similar diminishing activity of the native 1, 3 PDOR is perceived in *L. reuteri *HR2, along with elevated rates of lactate and ethanol production.

The enhanced formation rates of lactate and ethanol observed in the recombinant *L. reuteri *strain could be indirectly linked to the preferential utilization of NADPH by YqhD for 3-HPA conversion. The consumption of NADPH by YqhD and a possible reduction in the native NADH-dependent 1, 3-PDOR activity could have led to an increased cellular NADH/NAD^+ ^ratio. The surplus NADH thus generated has been diverted for the production of *NADH-consuming *metabolites like lactate and ethanol.

The elevated specific production rate of ethanol with concomitant decrease in specific acetate production rate implies that acetyl phosphate is channeled more towards ethanol production (Figure 5). This is most likely reflected as a shift in metabolism from acetate to ethanol production, resulting in reduced ATP synthesis. The decreased ATP production coupled with the diversion of NADPH away from biosynthesis by YqhD, could have contributed to the decreased growth rate of the recombinant culture (Jarboe et al. 2010; Zhu et al. 2009). The decreased μ_max _of the recombinant strain could also be attributed to the metabolic load imposed by the recombinant plasmid on the host (Bentley et al. 1990). Further, metabolic flux analysis needs to be carried out by measuring the enzyme activities and cofactors to verify this hypothesis. The present work has indicated that metabolic engineering can be effectively used to enhance 1, 3-PD productivity in *L. reuteri*. Further engineering of the strain to improve the redox balance and minimize the formation of byproducts like lactate and ethanol could pave the way for maximizing 1, 3-PD biosynthesis.

## Competing interests

The authors declare that they have no competing interests.
